# DGCR8 Promotes Neural Progenitor Expansion and Represses Neurogenesis in the Mouse Embryonic Neocortex

**DOI:** 10.3389/fnins.2018.00281

**Published:** 2018-04-30

**Authors:** Nadin Hoffmann, Stefan C. Weise, Federica Marinaro, Tanja Vogel, Davide De Pietri Tonelli

**Affiliations:** ^1^Neurobiology of miRNA Laboratory, Department of Neuroscience and Brain Technologies, Fondazione Istituto Italiano di Tecnologia, Genoa, Italy; ^2^Department of Molecular Embryology, Medical Faculty, Institute for Anatomy and Cell Biology, Albert-Ludwigs-University Freiburg, Freiburg, Germany

**Keywords:** corticogenesis, neurogenesis, DGCR8, DROSHA, microprocessor

## Abstract

DGCR8 and DROSHA are the minimal functional core of the Microprocessor complex essential for biogenesis of canonical microRNAs and for the processing of other RNAs. Conditional deletion of *Dgcr8* and *Drosha* in the murine telencephalon indicated that these proteins exert crucial functions in corticogenesis. The identification of mechanisms of DGCR8- or DROSHA-dependent regulation of gene expression in conditional knockout mice are often complicated by massive apoptosis. Here, to investigate DGCR8 functions on amplification/differentiation of neural progenitors cells (NPCs) in corticogenesis, we overexpress *Dgcr8* in the mouse telencephalon, by *in utero* electroporation (*IU*Ep). We find that DGCR8 promotes the expansion of NPC pools and represses neurogenesis, in absence of apoptosis, thus overcoming the usual limitations of *Dgcr8* knockout-based approach. Interestingly, DGCR8 selectively promotes basal progenitor amplification at later developmental stages, entailing intriguing implications for neocortical expansion in evolution. Finally, despite a 3- to 5-fold increase of DGCR8 level in the mouse telencephalon, the composition, target preference and function of the DROSHA-dependent Microprocessor complex remain unaltered. Thus, we propose that DGCR8-dependent modulation of gene expression in corticogenesis is more complex than previously known, and possibly DROSHA-independent.

## Introduction

Corticogenesis is a complex neurodevelopmental process leading to the formation of the cerebral cortex, the outer-most horizontally six-layered structure of the mammalian brain. This process requires the precise coordination of neural progenitor cell (NPC) proliferation, differentiation and migration (Taverna et al., 2014). The evolutionary expansion of the neocortex is tightly connected with the development of higher cognitive functions and consciousness in humans (Sun and Hevner, 2014). Expansion of the neocortex occurs in both the radial and lateral dimensions and it is due to an increase in the number of neurons and glial cells (Martínez-Cerdeño et al., 2006; Rakic, 2009; Borrell and Götz, 2014). This process is determined during development and primarily reflects the increase in the number of NPCs in the germinative layers of the dorsal telencephalon, the foremost region of the developing neural tube (Taverna et al., 2014). Thus, the number of NPC divisions and their switch from proliferative self-amplifying to neurogenic divisions is finely regulated in time and space, determining the size of the NPC pools during corticogenesis (Molyneaux et al., 2007; Taverna et al., 2014). Understanding the molecular mechanisms controlling the NPC pool size, temporal and spatial regulation of neurogenesis remain fundamental questions for developmental neurobiology, which entails important implications for neocortical expansion in evolution and for the pathophysiology of neurodevelopmental disorders.

The RNA binding protein DGCR8, encoded by the *DiGeorge syndrome critical region gene 8* (or *Pasha* in Drosophila), and type III ribonuclease (RNAse) protein DROSHA are the minimal functional core of the nuclear Microprocessor complex, essential for the biogenesis of canonical microRNAs (miRNA, Ha and Kim, 2014). In the last decade, conditional deletion of *Drosha, Dgcr8*, and other “miRNA-biogenesis” genes has been widely used to deplete mature miRNAs in corticogenesis *in vivo* (see for review Yang and Lai, 2011; Barca-Mayo and De Pietri Tonelli, 2014; Petri et al., 2014). This approach has contributed to elucidate the essential functions of these proteins during development of the central nervous system. However, it has also some disadvantages. For example, conditional knockout of *Drosha* or *Dgcr8*, in the developing nervous system often induces apoptosis entailing massive tissue derangement, complicating the interpretation of results (see for review Barca-Mayo and De Pietri Tonelli, 2014; Petri et al., 2014). Moreover, beside miRNA biogenesis, DROSHA and other “miRNA-pathway” proteins have additional RNA-processing functions (Burger and Gullerova, 2015). Indeed, DROSHA, DGCR8, and TAR DNA-binding protein 43 (TDP-43, another protein associated to the “Microprocessor” complex), also process messenger RNAs (mRNAs) encoding key transcription factors for neurogenesis, such as Neurogenin 2 (*Ngn2*), T-box brain 1 (*Tbr1*), and Nuclear factor 1 B (*NF1B*), silencing their expression independently of miRNAs (Knuckles et al., 2012; Di Carlo et al., 2013; Rolando et al., 2016; Marinaro et al., 2017). These alternative functions of miRNA-pathway proteins constitute a new post-transcriptional mechanism to control gene expression, which is still largely unexplored in neurogenesis.

We previously found, by phenotypic comparison of *Dgcr8* and *Dicer* conditional knockout mice, that miRNA-independent RNA processing functions of DGCR8 predominate over the miRNA-dependent ones in corticogenesis. In particular, *Dgcr8* deletion resulted in premature loss of NPCs, enhanced generation of TBR1+ neurons and induction of apoptosis leading to massive impairment of corticogenesis (Marinaro et al., 2017). However, the massive tissue derangement observed in the telencephalon of *Dgcr8* knockout mouse embryos, left unclear whether the premature neurogenesis observed in embryonic cortices of the mutants was due to DGCR8-dependent control of NPC fate, or a secondary effect due to loss of NPC polarity/delamination (Cappello et al., 2006; Arai and Taverna, 2017).

Here, to directly investigate DGCR8 functions on amplification/differentiation of NPCs in corticogenesis we overexpress *Dgcr8* in the mouse telencephalon, by *in utero* electroporation (*IU*Ep). Our results demonstrate that DGCR8 promotes the expansion of NPC pools and represses neurogenesis, possibly by promoting NPC proliferation. Moreover, we found that overexpression of DGCR8 in embryonic mouse neocortex does not alter the molecular composition of the “DROSHA-Microprocessor” complex or its preference for targets, suggesting the existence of multiple DGCR8-dependent mechanisms to regulate corticogenesis.

## Results

### Overexpression of DGCR8 in the mouse telencephalon alters the relative distribution of cells across the cortical wall in absence of apoptosis

**Figure 1:**
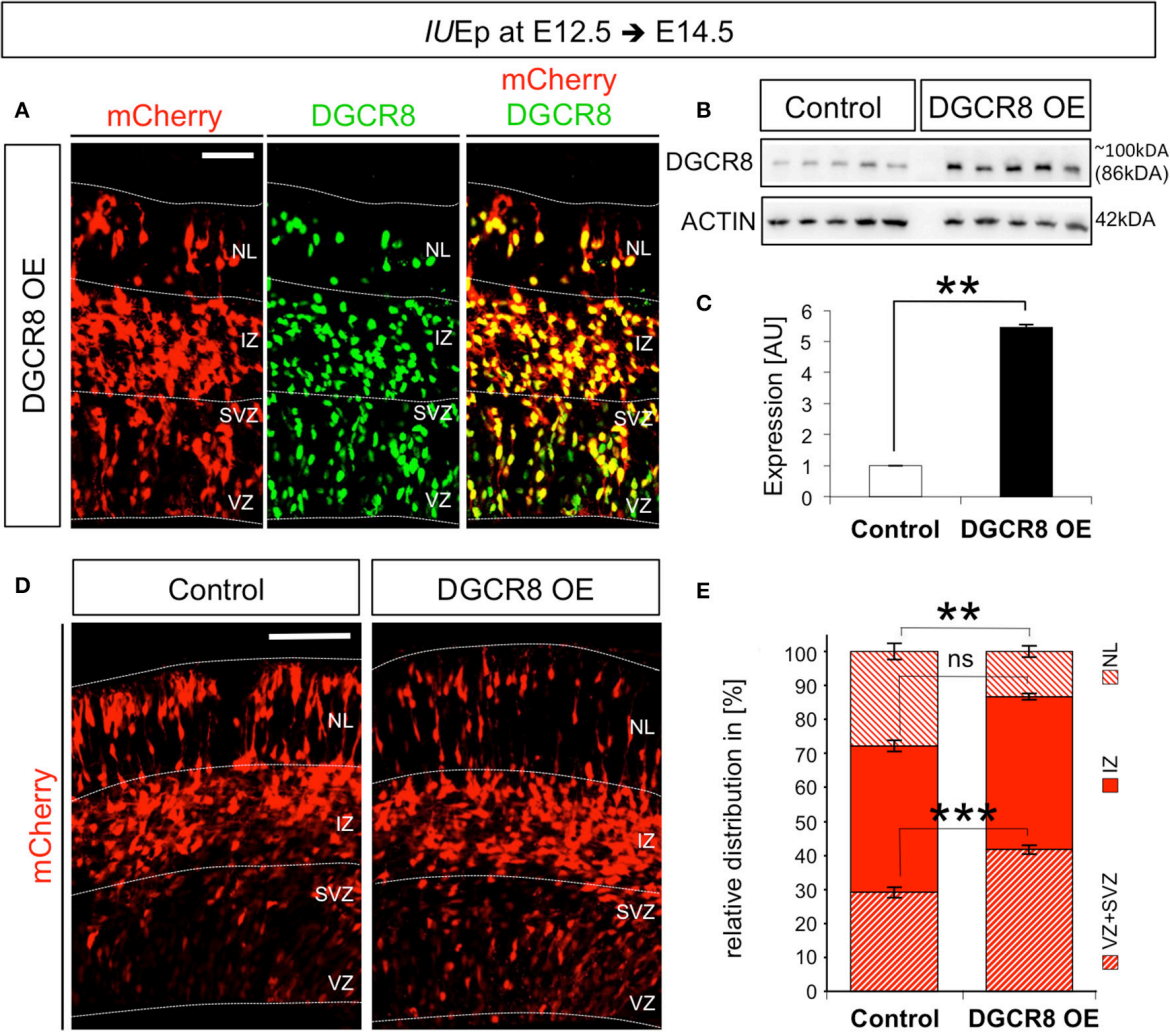
Overexpression of DGCR8 in the mouse telencephalon alters the relative distribution of cells across the cortical wall **(A)** Immunofluorescence staining for DGCR8 and intrinsic mCherry fluorescence in coronal cryosections through the dorsal telencephalon of mouse embryos at E14.5 overexpressing DGCR8 **(B,C)**, after *IU*Ep at E12.5. **(B)** Western blot of DGCR8, and **(C)** Quantification of DGCR8 protein level in the telencephalon of E14.5 mice electroporated at E12.5 with pCAGGS-mCherry (Control, white bar, 5 independent pools shown) or pCAGGS-mCherry and pCAGGS-*Dgcr8* plasmids (DGCR8 OE, black bar, five independent pools shown). Values are normalized on ACTIN. Error bars indicate the variation of five Control and five DGCR8 OE independent pools (s.e.m.); each independent pool consists of four to five dissected electroporated cortical areas; unpaired Student's *t*-test. **(D)** Immunofluorescence microscopy of coronal cryosections through the telencephalon at E14.5 after *IU*Ep at E12.5 showing intrinsic mCherry fluorescence (red), as reporter of targeted cells. Dashed lines indicate borders of specific brain areas (from outside to inside: NL: neuronal layer, IZ: intermediate zone, SVZ: subventricular zone and VZ: ventricular zone), scale bar: 100 μm. **(E)** Quantification of the relative distribution of electroporated cells in NL, IZ, and SVZ+VZ expressed in % over total mCherry+ cells; Error bars indicate the variation of four Control and five DGCR8 OE electroporated cortices (s.e.m.); unpaired Student's *t*-test. **p-value < 0.01; ***p-value < 0.001.

To overexpress *Dcgr8* in NPCs and their differentiated progeny we used *in utero* electroporation (*IU*Ep, De Pietri Tonelli et al., 2006). By this means, we delivered pCAGGS-mCherry plasmid into the dorsal telencephalon of E12.5 wild-type (WT) mouse embryos (Figure 1, Control), or pCAGGS-mCherry along with a plasmid constitutively expressing mouse DGCR8 (pCAGGS-mmu-*Dgcr8*, Figure 1 DGCR8 OE). Immunofluorescence analysis, performed at E14.5 (i.e., 48 h after co-electroporation), revealed that almost all the targeted cells (97 ± 0.2%; *n* = 3) overexpressed mCherry and DGCR8 proteins (when both plasmids were co-electroporated, Figure 1A), compared to the endogenous DGCR8 levels (Figure S1, control cortices and mCherry negative cells in DGCR8 OE cortices). Analysis of protein extracts from the electroporated cortices by western blotting confirmed a significant 5-fold increase of DGCR8 expression, compared to control cortices (Figures 1B,C, DGCR8 OE vs. Control, *n* = 5 independent experiments shown; Original Immunoblot in Figure S3).

To investigate effects of the DGCR8 overexpression on fate of the targeted cells, we analyzed the distribution of mCherry+ cells across the cortical wall at E14.5. Overexpression of DGCR8 led to a significant decrease in the proportion of targeted cells located in the neuronal layers (NL) and an increase in the proportion of targeted cells in the progenitor layers [i.e., the Ventricular Zone (VZ) and Subventricular Zone (SVZ)] compared to control cortices (Figures 1D,E, DGCR8 OE vs. Control). Whereas the proportion of targeted cells in the intermediate zone (IZ) remained unaltered in both conditions (Figures 1D,E).

**Figure 2:**
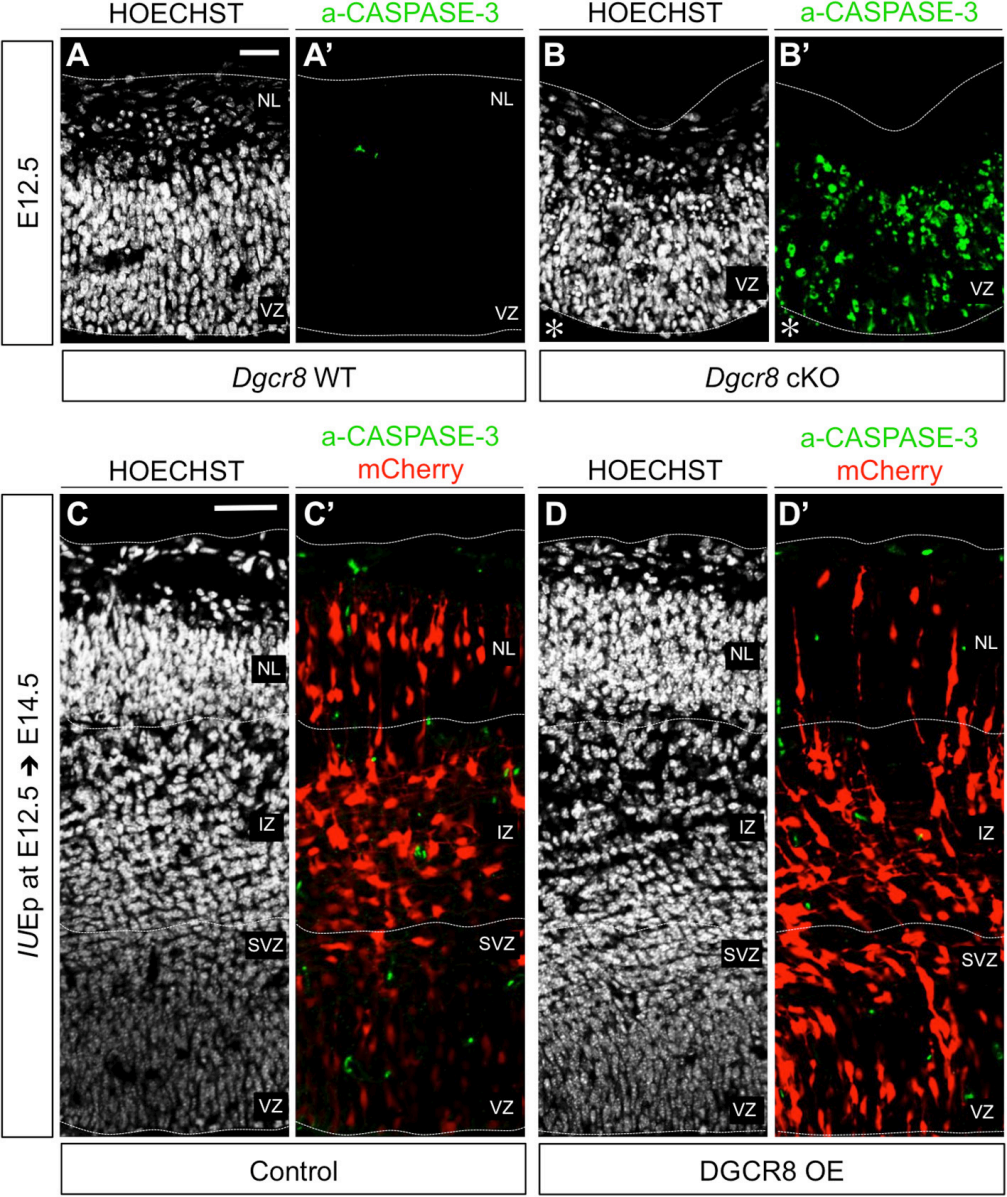
Overexpression of DGCR8 does not lead to apoptosis at E14.5 **(A–D)** Hoechst staining on coronal cryosections through the dorsal telencephalon of *Dgcr8* WT **(A)** and *Dgcr8* conditional knockout (cKO) **(B)** mouse embryos at E12.5 or on coronal cryosections through the dorsal telencephalon of Control **(C)** and DGCR8 OE **(D)** mouse embryos at E14.5 after *IU*Ep at E12.5. **(A'–D')** Immunostaining for activated CASPASE-3 (green) on coronal cryosections through the dorsal telencephalon of *Dgcr8* WT **(A')** and *Dgcr8* cKO **(B')** mouse embryos at E12.5 or on coronal cryosections through the dorsal telencephalon of Control **(C')** and DGCR8 OE **(D')** mouse embryos at E14.5 after *IU*Ep at E12.5; electroporated cells (mCherry, red); dashed lines indicate limits of the cortical wall (from outside to inside: NL: neuronal layer, IZ: intermediate zone, SVZ: subventricular zone and VZ: ventricular zone); scale bars: 20 μm **(A–B')**; scale bar: 100 μm **(C–D')**. *p-value < 0.05; **p-value < 0.01.

We previously found that conditional deletion of *Dgcr8* during corticogenesis induces apoptosis leading to a massive disorganization of the developing cortex (Marinaro et al., 2017). Here, to ascertain whether the reduced proportion of cells in NL upon overexpression of DGCR8 (Figure 1) was due to cell loss, we analyzed electroporated cortices for apoptosis (Figure 2 and Figure S2). Sections through cortices of E12.5 and E13.5 conditional *Dgcr8* knockout (*Dgcr8* cKO) mice (Marinaro et al., 2017) were used as positive control for apoptosis. As expected, apoptotic cells were observed in these cortices as revealed by pyknotic nuclei and by immunofluorescence staining for activated CASPASE-3 (Figure 2 and Figures S2B,B', *Dgcr8* cKO), compared to cortices from WT littermates (Figure 2 and Figures S2A,A', *Dgcr8* WT). In contrast, overexpression of DGCR8 did not induce apoptosis either at E13.5 (i.e., 24 h after electroporation Figures S2C–D'), or at E14.5, (i.e., 48 h after electroporation, Figures 2D,D', DGCR8 OE), compared to control-electroporated cortices (Figures 2C,C', Control).

These results indicate that overexpression of DGCR8 impairs accumulation of cells in the NL in absence of cell death, while it promotes retention of cells in the VZ/SVZ. This suggests that DGCR8 function might promote self-renewal of NPCs and repress differentiation and/or migration of newborn cortical projection neurons.

### Overexpression of DGCR8 decreases the generation of deep-layer neurons

**Figure 3:**
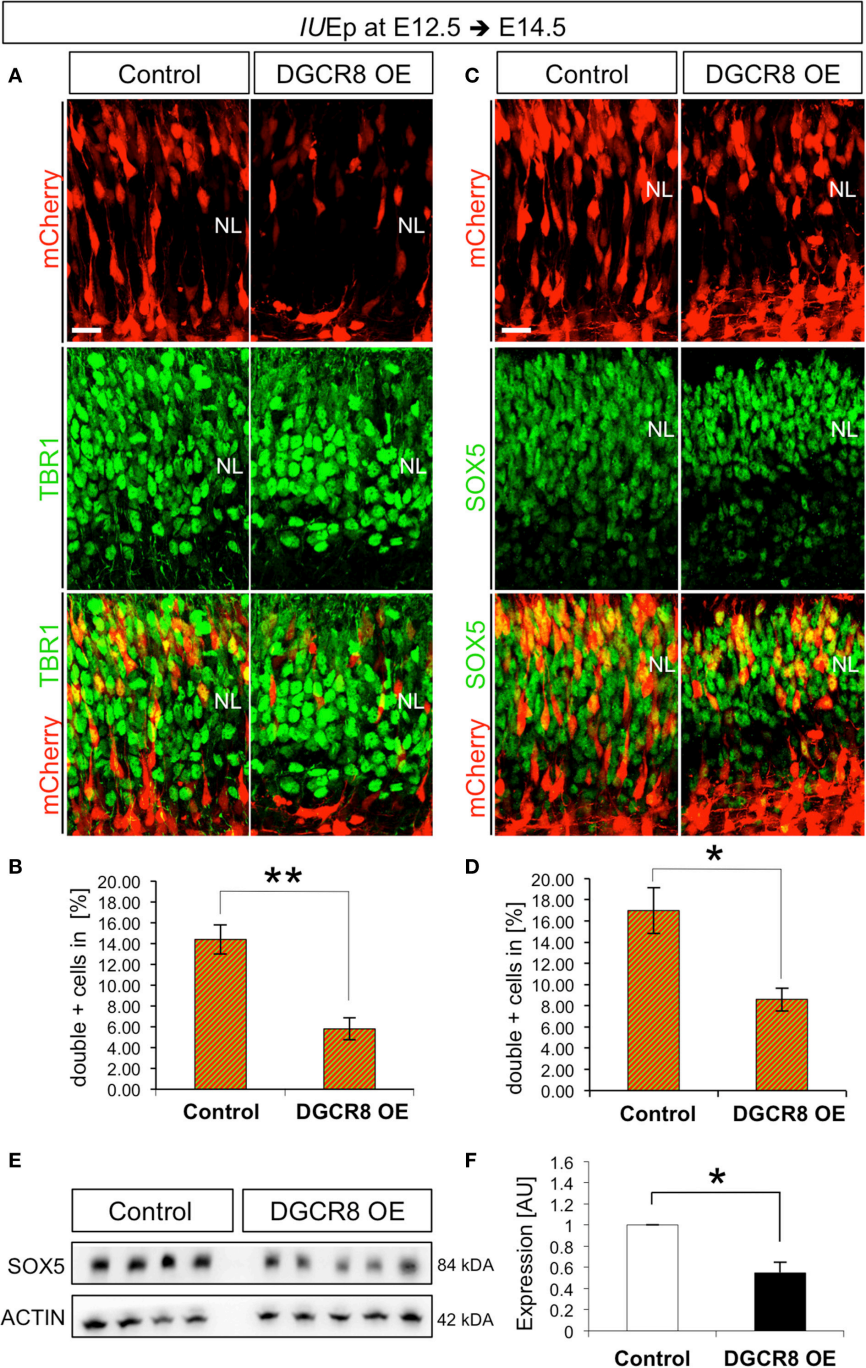
Overexpression of DGCR8 decreases the generation of deep-layer neurons **(A–D)** Immunostaining for TBR1 (**A**, green) or SOX5 (**C**, green) and mCherry+ electroporated cells (**A,C**, red) and merged images on coronal cryosections through the dorsal telencephalon of Control and DGCR8 OE mouse embryos at E14.5 after *IU*Ep at E12.5, and quantification of the proportion of TBR1+mCherry+ **(B)** or SOX5+mCherry+ **(D)** cells expressed in % over total mCherry+ cells. NL: neuronal layer; scale bar: 20 μm. Error bars indicate the variation of four Control and five DGCR8 OE electroporated cortices (s.e.m.); unpaired Student's *t*-test. **E,F**) Western blot and quantification of SOX5 [four (Control) and five (DGCR8 OE) independent pools] in E14.5 Control (white bar) and DGCR8 OE (black bar) mouse dorsal telencephalon after *IU*Ep at E12.5. Values are normalized on ACTIN levels. Error bars indicate the variation of four Control and four DGCR8 OE independent pools (s.e.m.); each independent pool consists of four to five dissected electroporated cortical areas; unpaired Student's *t*-test.

*IU*Ep has been previously used for birth-dating and fate analysis of newborn cells in the mouse neocortex, indicating that the majority of targeted NPCs at E12.5 give rise to neurons that populate cortical deep-layer VI (Langevin et al., 2007). To investigate whether the reduction of cells accumulating in the NL upon DGCR8 overexpression (Figure 1) was due to reduced generation and/or migration of deep-layer neurons, we quantified the proportion of targeted (mCherry+) cells that were also positive for TBR1, a transcription factor known to be expressed and to specify mostly deep-layer neurons (Hevner et al., 2001), across the entire cortical wall at E14.5 (Figure 3A). Overexpression of DGCR8 reduced the proportion of TBR1+mCherry+ double-positive deep-layer neurons (Figures 3A,B, DGCR8 OE vs. Control).

We previously showed that TBR1 is post-transcriptionally repressed by DGCR8 (Marinaro et al., 2017), questioning its reliability as marker of deep-layer neurons upon DGCR8 overexpression. Thus, to ascertain whether the decrease in TBR1+mCherry+ double-positive cells reflected a reduction in TBR1 expression, or the generation of deep-layer VI neurons, we investigated by immunofluorescence staining the expression of another transcription factor, Sex Determining Region Y-Box 5 (SOX5), known to be involved in deep-layer VI neuron specification (Arlotta et al., 2005). Indeed, overexpression of DGCR8 also decreased the proportion of SOX5+mCherry+ double-positive deep-layer neurons compared to control (Figures 3C,D, DGCR8 OE vs. Control). Next, we corroborated these results by analysis of protein extracts from the electroporated cortices, confirming a significant decrease in the expression of SOX5 upon DGCR8 overexpression (Figures 3E,F, DGCR8 OE vs. Control, *n* = 4 (Control) and *n* = 5 (DGCR8 OE) independent experiments shown; Original Immunoblot in Figure S3). Given that DGCR8 overexpression reduces the generation of TBR1+ neurons (this study), while we previously found that depletion of *Dgcr8* increased it (Marinaro et al., 2017), collectively this evidence supports a function of DGCR8 to regulate neurogenesis in the embryonic mouse neocortex.

### Overexpression of DGCR8 promotes NPC expansion

DGCR8 overexpression reduced the generation of neurons (Figures 1, 3) while it increased the proportion of targeted cells retained in VZ/SVZ (Figure 1). Thus, we hypothesized that DGCR8 might decrease neurogenesis by promoting self-amplification of NPCs.

In the murine telencephalon, the two principal classes of NPCs can be identified by their location during mitosis and expression of specific markers (Taverna et al., 2014). In particular, neuroepithelial, radial glia cells and short neural precursors (from here collectively defined as “Apical Progenitors,” APs) are elongated epithelial cells that divide at the ventricular surface, and express the transcription factor Paired Box gene 6 (PAX6) (Götz and Barde, 2005). APs generate other types of NPCs, such as basal intermediate progenitors (from here defined as “Basal Progenitors,” BPs). BPs delaminate from the neuroepithelium, express the transcription factor T-Box Brain Protein 2 (TBR2, or Eomes, Englund et al., 2005) and divide at the basal side of the VZ and in the SVZ, becoming the predominant neurogenic type from E14.5 (Haubensak et al., 2004; Miyata et al., 2004; Noctor et al., 2004).

**Figure 4:**
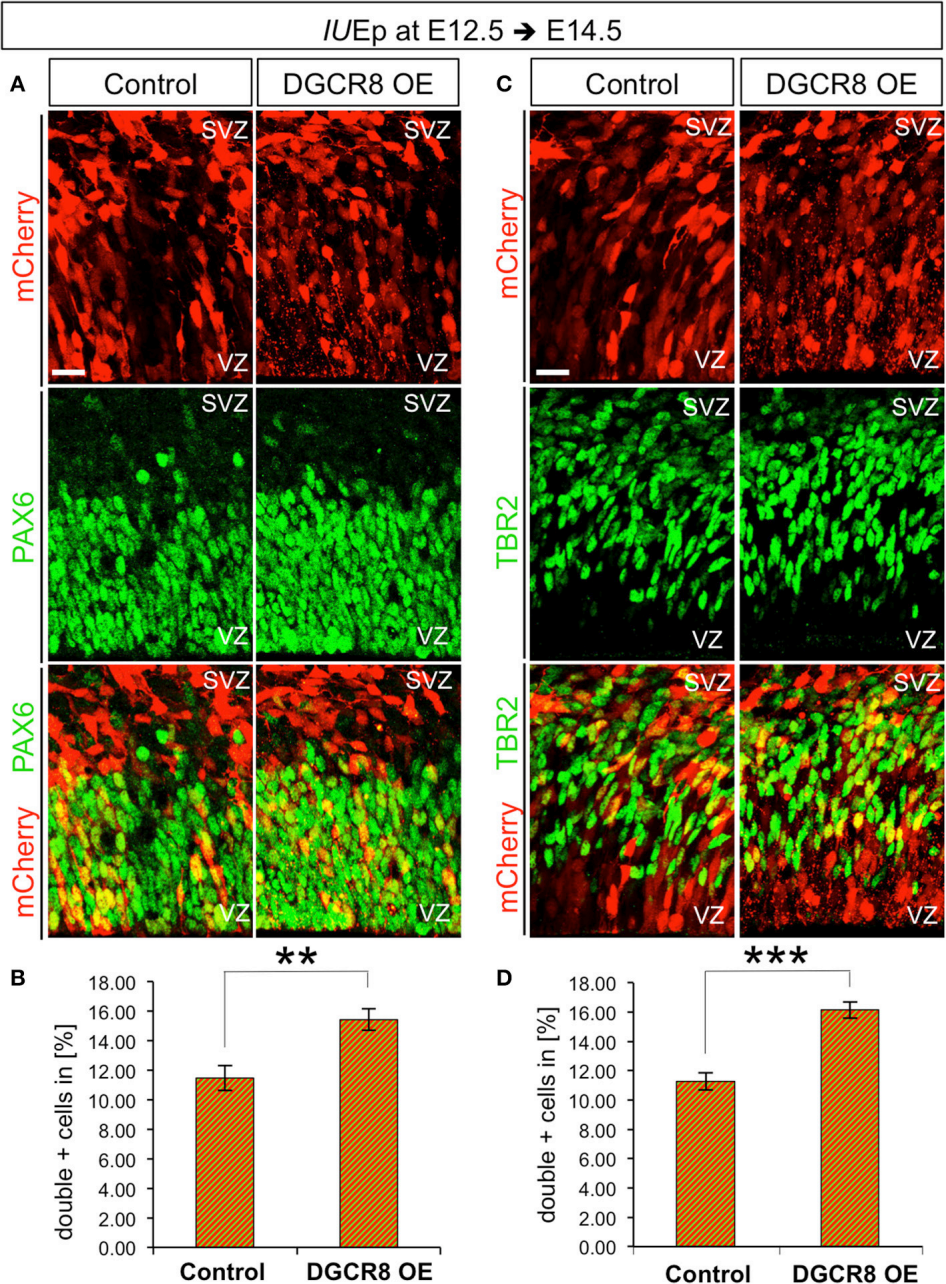
Overexpression of DGCR8 promotes NPC expansion **(A–D)** Immunostaining for PAX6 (**A**, green) or TBR2 (**C**, green) and mCherry+ electroporated cells (**A,C**, red) and merged images on coronal cryosections through the dorsal telencephalon of Control and DGCR8 OE mouse embryos at E14.5 after *IU*Ep at E12.5, and quantification of the proportion of PAX6+ mCherry+ **(B)** or TBR2+ mCherry+ **(D)** cells expressed in % over total mCherry+ cells. SVZ: subventricular zone and VZ: ventricular zone: scale bar: 20 μm. Error bars indicate the variation of five Control and four DGCR8 OE electroporated cortices (s.e.m.); unpaired Student's *t*-test. **p-value < 0.01; ***p-value < 0.001.

To investigate the effects of DGCR8 manipulation in APs and BPs, we analyzed electroporated cortices (as in Figure 1) by immunofluorescence staining for PAX6 and TBR2 and quantified proportions of PAX6+mCherry+ APs and TBR2+mCherry+ BPs at E14.5 (Figure 4). DGCR8 overexpression led to a significant increase in the proportion of both PAX6+mCherry+ APs (Figures 4A,B, DGCR8 OE vs. Control) and TBR2+ mCherry+ BPs (Figures 4C,D, DGCR8 OE vs. Control) compared to control. These results indicate that DGCR8 promotes amplification of the two major subtypes of cortical NPCs, during early corticogenesis.

### Overexpression of DGCR8 promotes NPC proliferation

**Figure 5:**
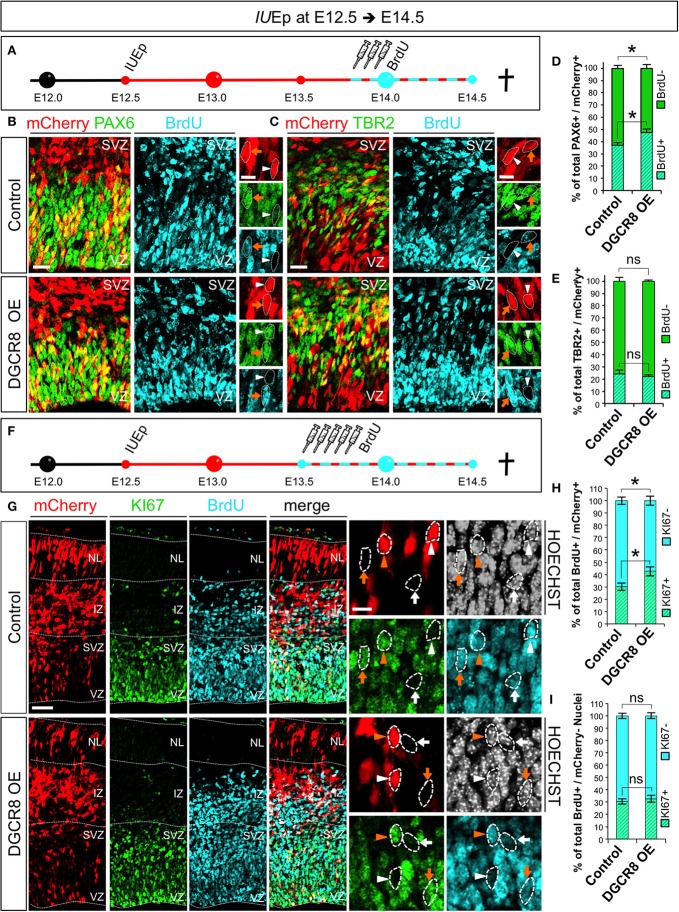
Overexpression of DGCR8 stimulates NPC proliferation and cell cycle re-entry **(A)** Schematic representation of *in utero* electroporation and 10 h BrdU pulse/chase experiment. **B,C**) Co-Immunostaining for PAX6 (**B**, green) or TBR2 (**C**, green), BrdU (**B,C**, cyan) and mCherry+ electroporated cells (**B,C** red) on coronal cryosections through the dorsal telencephalon of Control and DGCR8 OE mouse embryos at E14.5 after *IU*Ep at E12.5. **D,E**) Quantification of the proportion of mCherry+PAX6+ that were BrdU+, or BrdU− **(D)**; or mCherry+TBR2+ that were BrdU+ or BrdU− **(E)** cells, expressed in % over total mCherry+PAX6+(or mCherry+TBR2+) cells in a selected area (i.e., VZ+SVZ); scale bar: 20 and 10 μm in high magnification images; white arrowheads: mCherry+ and PAX6+ or TBR2+ cells that are BrdU−, orange arrows: mCherry+ and PAX6+ or TBR2+ cells that are BrdU+. Error bars indicate the variation of five Control and six DGCR8 OE electroporated cortices (s.e.m.); unpaired Student's *t*-test. **(F)** Schematic representation of *in utero* electroporation and 24 h BrdU pulse/chase experiment. **(G)** Co-Immunostaining for KI67 (green), BrdU (cyan), mCherry+ electroporated cells (red), and Nuclei (Hoechst, gray) on coronal cryosections through the dorsal telencephalon of Control and DGCR8 OE mouse embryos at E14.5 after *IU*Ep at E12.5. **(H)** Quantification of the proportion of KI67+ (cell cycle re-entry) or KI67− (cell cycle exit) BrdU+ mCherry+ cells expressed in % over total BrdU+ mCherry+ cells across the whole cortical wall. **(I)** Quantification of the proportion of KI67+, or KI67−, BrdU+ mCherry− cells expressed in % over total BrdU+ mCherry– cells (identified by Hoechst) across the whole cortical wall (same ROI as in **H**); VZ: ventricular zone, SVZ: subventricular zone and NL: neuronal layer; scale bar: 100 and 10 μm in high magnification images; white arrowheads: mCherry+ and BrdU+ cells that are KI67−, orange arrowheads: mCherry+ and BrdU+ cells that are KI67+, white arrows: mCherry– BrdU+ cells that are KI67− and orange arrows: mCherry− BrdU+ cells that are KI67+. Error bars indicate the variation of five Control and five DGCR8 OE electroporated cortices (s.e.m.); unpaired Student's *t*-test. *p-value < 0.05.

Next, we asked whether DGCR8 overexpression promotes NPC expansion by stimulating their proliferation (Figure 5). Cell cycle is one of the key determinants of the NPC amplification and differentiation in corticogenesis (Dehay and Kennedy, 2007) and proliferating NPCs have a shorter cell cycle compared to neurogenic NPCs (Caviness et al., 1995; Takahashi et al., 1995). We electroporated mCherry (Control) and mCherry/*Dgcr8* (DGCR8 OE) at E12.5 and investigated proliferation of NPCs at E14.5, upon administration of Bromodeoxyuridine (BrdU) pulses (three, every 2 h) over 10 h (Figure 5A). Overexpression of DGCR8 increased BrdU incorporation in PAX6+mCherry+ APs (Figures 5B,D, DGCR8 OE vs. Control), compared to control. In contrast, analysis of BrdU+TBR2+mCherry+ BPs did not reveal significant differences between cortices electroporated with DGCR8 or control (Figures 5C,E, DGCR8 OE vs. Control).

Next, we investigated cell cycle re-entry and exit of NPCs at E14.5. After electroporation of mCherry (Control) or mCherry/*Dgcr8* (DGCR8 OE) at E12.5, we administered Bromodeoxyuridine (BrdU) pulses (five every 2 h, Figure 5F). Twenty-four hours later we repeated analysis of TBR2+ BPs for proportions of BrdU positive or negative staining and again we did not find differences between Control or DGCR8 OE cortices in this experimental setting (data not shown). Next, we immuno-stained sections from these electroporated cortices with antibodies anti-KI67 (a protein that is expressed in all phases of the cell cycle except G0 and early G1, Yu et al., 1992) and BrdU (Figure 5G, quantification in Figure 5H). In cortices overexpressing DGCR8, we found a ~10% increase of mCherry and BrdU double-positive cells that were also KI67+ (cell cycle re-entry) and an equivalent decrease in mCherry and BrdU double-positive cells that were KI67– (cell cycle exit), compared to control cortices (Figures 5G,H; DGCR8 OE vs. Control). Importantly, the proportion of cell cycle re-entry and of exit in non-electroporated cells (mCherry–) remained similar in both conditions (Figure 5G, Hoechst+ mCherry– cells in both Control and DGCR8 OE cortices, quantification in Figure 5I). These results suggest that overexpression of DGCR8 cell-autonomously promotes the expansion of NPC pools by stimulating their proliferation. Our observations are consistent with evidence indicating that DGCR8 is required for normal proliferation and cell-cycle progression of embryonic stem cells (ESCs) (Wang et al., 2007) and with our previous data in NPCs of the *Dgcr8* cKO cortices (Marinaro et al., 2017).

### Overexpression of DGCR8 decreases the generation of upper-layer neurons and promotes BP expansion at E16.5

**Figure 6:**
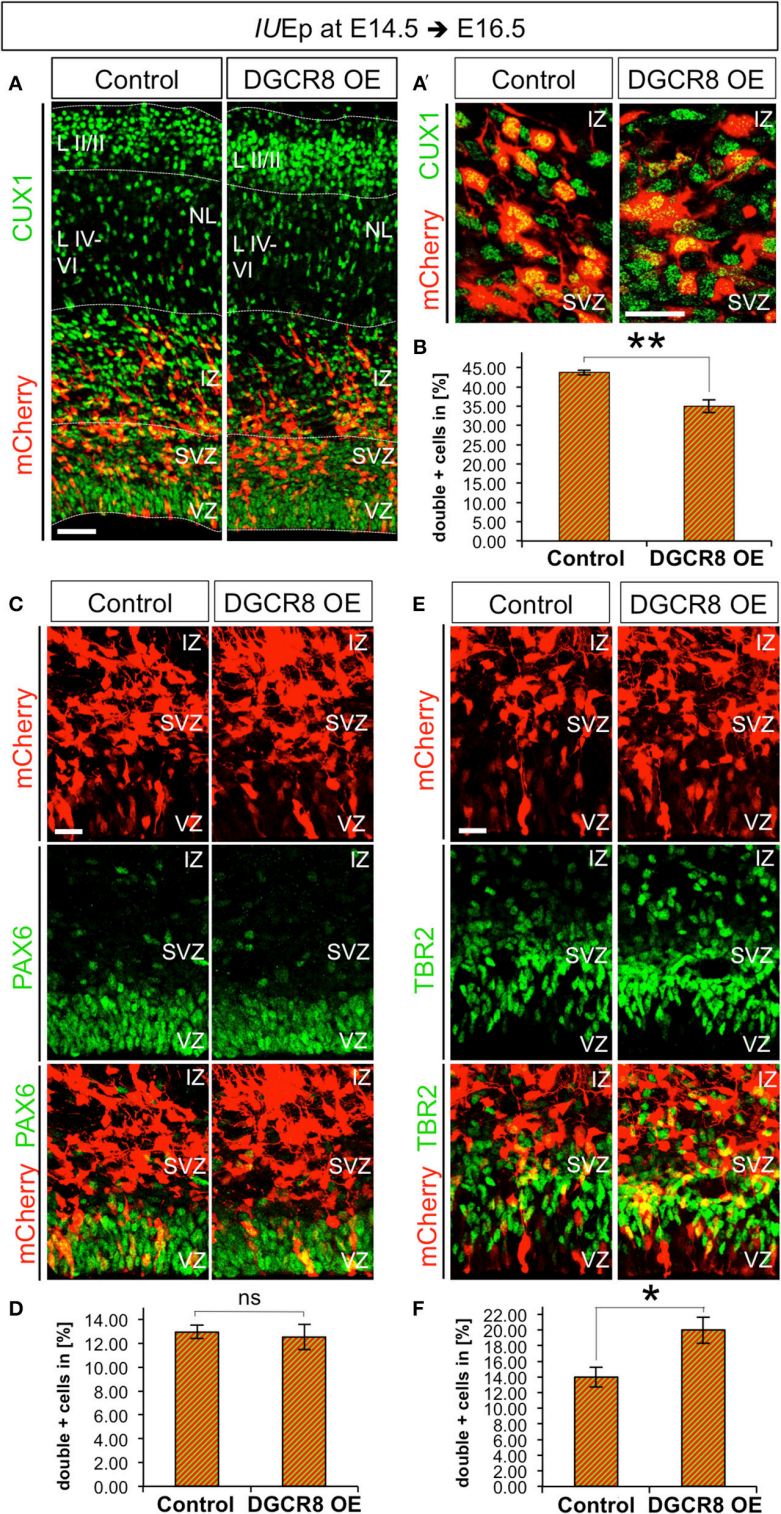
Overexpression of DGCR8 decreases the generation of upper-layer neurons and selectively promotes BP expansion at E16.5 **(A,A')** Immunostaining for CUX1 (green) and mCherry+ electroporated cells (red) on coronal cryosections through the dorsal telencephalon of Control and DGCR8 OE mouse embryos at E16.5 after *IU*Ep at E14.5. **(B)** Quantification of the proportion of CUX1+ mCherry+ **(A,A')** cells expressed in % over total mCherry+ cells; scale bar 50 μm **(A)** and 20 μm **(A')**. VZ: ventricular zone, SVZ: subventricular zone, IZ: intermediate zone, NL: neuronal layer, LII-III: cortical layer 2 and 3, LIV-VI: cortical layer 4-5. Error bars indicate the variation of four Control and four DGCR8 OE electroporated cortices (s.e.m.); unpaired Student's *t*-test. **(C,E)** Immunostaining for PAX6 (**C**, green) or TBR2 (**E**, green) and mCherry+ electroporated cells (**C,E**, red) and merged images on coronal cryosections through the dorsal telencephalon of Control and DGCR8 OE mouse embryos at E16.5 after *IU*Ep at E14.5. **D,F**) Quantification of the proportion of PAX6+ mCherry+ **(D)** or TBR2+ mCherry+ **(F)** cells expressed in % over total mCherry+ cells; scale bar: 20 μm. Error bars indicate the variation of five Control and five DGCR8 OE electroporated cortices (s.e.m.); unpaired Student's *t*-test. *p-value < 0.05; **p-value < 0.01.

We aimed to investigate whether DGCR8 functions change at later stages of corticogenesis. Thus, we repeated the electroporation experiments at E14.5 and analyzed brains at E16.5 (i.e., 48 h after electroporation), a stage in which NPCs mostly generate neurons of cortical layers II to IV (Langevin et al., 2007). Layer II-IV neurons can be identified by immunofluorescence staining for Cut-Like Homeobox 1 (CUX1) a transcription factor that is already expressed by VZ/SVZ progenitors from which these neurons originate (Nieto et al., 2004). Quantification of CUX1 staining at E16.5 revealed that overexpression of DGCR8 led to a significant decrease in the proportion of targeted cells (mCherry+) that were also CUX1+ (Figures 6A,A',B, DGCR8 OE vs. Control), compared to control-electroporated cortices. Next, to investigate the effects of DGCR8 manipulation in APs and BPs at this stage of development we quantified proportions of PAX6+mCherry+ APs and TBR2+mCherry+ BPs (Figures 6C–F). This analysis revealed that overexpression of DGCR8 led to a selective increase in the proportion of TBR2+mCherry+ BPs (Figures 6E,F, DGCR8 OE vs. Control) but not of PAX6+mCherry+ APs (Figures 6C,D, DGCR8 OE vs. Control), compared to control. Analysis of BrdU incorporation in TBR2+ BPs at E16.5 (five BrdU pulses every 2 h and analysis 11 h later) again did not reveal any difference upon overexpression of DGCR8 (data not shown). Together, these results are consistent with previous findings indicating BPs as major source of upper-layer neurons (Taverna et al., 2014), and remarkably, they suggest that DGCR8 function might differently affect AP and/or BP expansion in corticogenesis at specific developmental times.

### Overexpression of DGCR8 does not alter composition or functions of the “miRNA-independent microprocessor”

We previously found that miRNA-independent RNA processing functions of DGCR8 predominate over miRNA-dependent ones in corticogenesis (Marinaro et al., 2017). Indeed, DROSHA (Knuckles et al., 2012) and DGCR8 (Marinaro et al., 2017) have been recently shown to regulate embryonic neurogenesis through miRNA-independent processing of *Ngn2* and *Tbr1* mRNAs. DROSHA and DGCR8 are essential components of the “miRNA-Microprocessor” complex (Denli et al., 2004; Gregory et al., 2004; Han et al., 2004; Landthaler et al., 2004), but the molecular components of “miRNA-independent Microprocessor” in cortical NPCs and neurons are currently unknown. Indeed, the Microprocessor is a dynamic complex and several proteins have been found to associate with DROSHA and regulate its function, such as TDP-43 (Di Carlo et al., 2013), DEAD-box helicase 5 (DDX5) (Buratti et al., 2010; Kawahara and Mieda-Sato, 2012; Di Carlo et al., 2013; Dardenne et al., 2014; Jung et al., 2014), SMAD protein signal transducers of the TGFbeta/BMP pathways (Davis et al., 2010), TLX, homolog of the Drosophila tailless gene homolog of the Nuclear receptor subfamily 2 group E member 1 gene (Murai et al., 2016) and Forkhead box protein G1 (FOXG1, SCW and TV personal communication), a transcription factor critical for forebrain development (Siegenthaler et al., 2008) and several others (see also Shiohama et al., 2007).

We hypothesized that overexpression of DGCR8 might alter the molecular composition of the Microprocessor, thereby shifting preference/cleavage efficiency of this complex for target RNAs. Alternatively, as DGCR8 can bind RNA through its RNA-binding domains independently from DROSHA (Nguyen et al., 2015), another possibility is that overexpression of DGCR8 might sequester RNA targets preventing their cleavage by DROSHA-complex and/or eventually directly modulating target expression, independently of DROSHA-complex.

**Figure 7:**
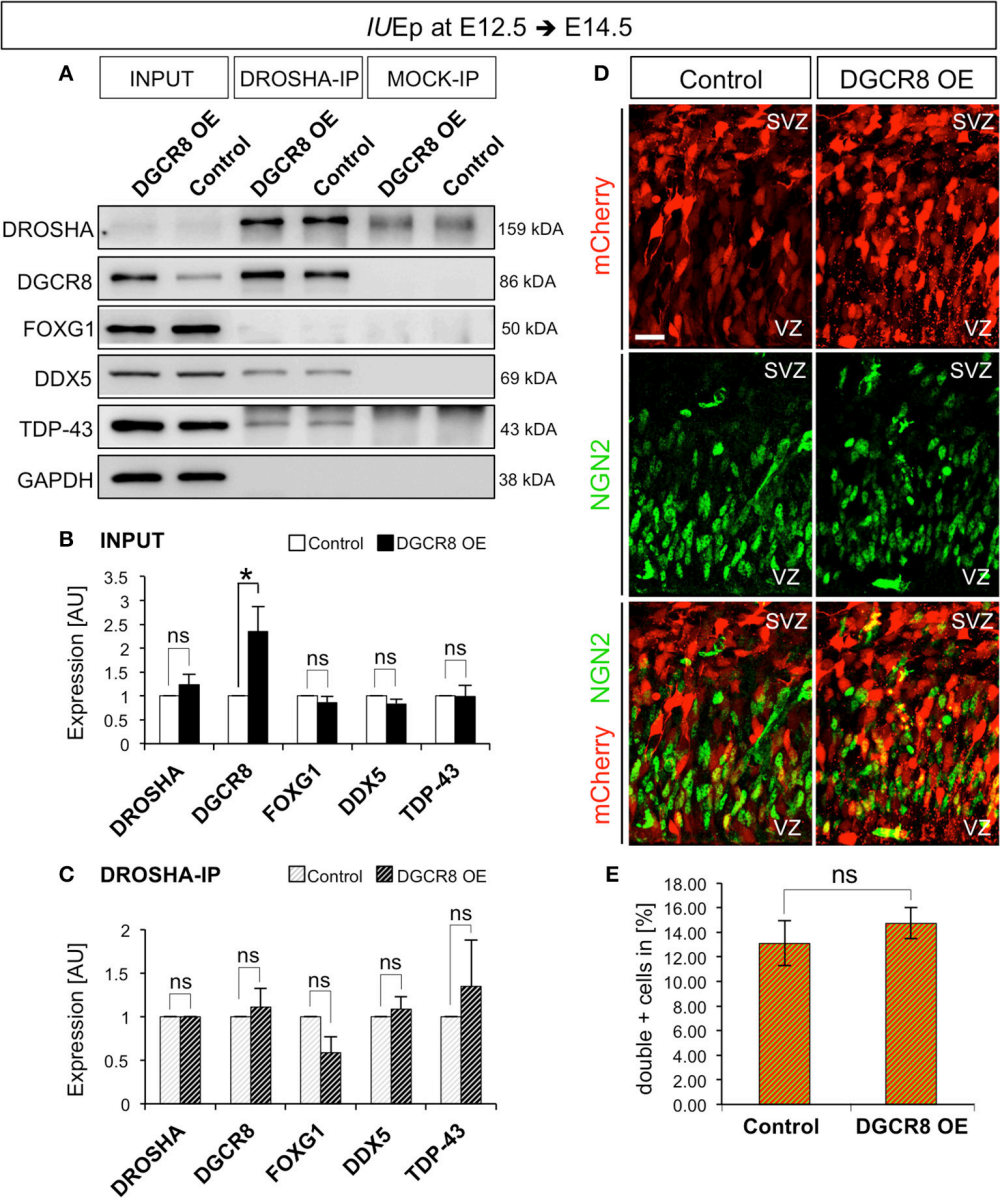
Overexpression of DGCR8 does not change composition or functions of the “miRNA-independent Microprocessor” **(A–C)** Western blot **(A)** and quantification **(B,C)** of DROSHA, DGCR8, FOXG1, DDX5 and TDP-43, in lysate (INPUT) or lysate after co-immunoprecipitation (co-IP) with DROSHA, or MOCK immunoprecipitation, from E14.5 Control (white bars, in **B**, or white-gray-striped bars, in **C**) and DGCR8 OE (black bars, in **B**, or black-gray-striped bars, in **C**) mouse dorsal telencephalon after *IU*Ep at E12.5. Samples were normalized over GAPDH for input samples and normalized to DROSHA for co-immunoprecipitation, error bars indicate the variation of four Control and four DGCR8 OE independent pools (s.e.m.); each independent pool consists of five to six dissected electroporated cortical areas; unpaired Student's *t*-test. **(D)** Immunostaining for NGN2 (green) and mCherry+ electroporated cells (red) on coronal cryosections through the dorsal telencephalon of Control and DGCR8 OE mouse embryos at E14.5 after *IU*Ep at E12.5. SVZ: subventricular zone and VZ: ventricular zone; scale bar: 20 μm. **(E)** Quantification of the proportion of NGN2+mCherry+ cells expressed in % over total mCherry+ cells; Error bars indicate the variation of four Control and four DGCR8 OE electroporated cortices (s.e.m.); unpaired Student's *t*-test. *p-value < 0.05.

To discriminate between these possibilities, we investigated the composition of the DROSHA-Microprocessor complex *in vivo* upon overexpression of DGCR8 (as in Figure 1), by immunoprecipitation (IP) of DROSHA, followed by analysis of co-IP proteins in protein extracts from electroporated cortices (Figure 7 and Original Immunoblot in Figure S4). Overexpression of DGCR8 did not alter the total levels of DROSHA, FOXG1, DDX5, and TDP-43 (Figure 7A, first two lanes, and B, INPUT, DGCR8 OE vs. Control), suggesting that the expression of these proteins is not controlled by DGCR8 in our condition. Surprisingly, overexpression of DGCR8 also did not alter levels of the proteins that co-precipitated with DROSHA (Figures 7A,C, DROSHA-IP, DGCR8 OE vs. Control), compared to control cortices, or mock IP (Figure 7A). This result suggests that the phenotypes observed upon DGCR8 overexpression in embryonic mouse neocortex are not due to changes in the molecular composition of the Microprocessor, with regard to the proteins considered in our analysis.

*Ngn2* mRNA, encoding a transcription factor involved in a sequential transcriptional cascade during corticogenesis (PAX6>NGN2>TBR2>TBR1) (Englund et al., 2005), is repressed by DROSHA independent of miRNAs (Knuckles et al., 2012) and DGCR8 is dispensable for the DROSHA-dependent processing of *Ngn2* mRNA (Di Carlo et al., 2013; Marinaro et al., 2017). To investigate whether overexpression of DGCR8 alters the RNA target preference or cleavage efficiency of the “miRNA-independent Microprocessor,” we analyzed the proportion of NGN2+ cells in the electroporated cortices by immunofluorescence staining. We found that overexpression of DGCR8 in the embryonic mouse neocortex does not alter NGN2 proportions (Figures 7D,E, DGCR8 OE vs. Control). This result indicates that overexpression of DGCR8 does not change target preference, or cleavage efficiency of the “miRNA-independent Microprocessor.” Thereby, this evidence opens the possibility that DGCR8 might achieve a direct post-transcriptional control of its targets, as previously proposed for TDP-43—and DROSHA—mediated repression of *Ngn2* translation (Knuckles et al., 2012; Di Carlo et al., 2013).

## Discussion

By overexpression of DGCR8 in the embryonic mouse neocortex, our study demonstrates that DGCR8 promotes the expansion of NPC pools and represses neurogenesis, possibly by a cell-autonomous mechanism. Interestingly, DGCR8 selectively promotes BP expansion at later developmental stages. With regard to the proteins and developmental time considered in our study, composition, target preference and functions of the “miRNA-independent Microprocessor” complex remained unaltered upon DGCR8 overexpression, suggesting that DGCR8-dependent control of gene expression in corticogenesis is more complex than previously known.

Previous studies, where *Drosha* or *Dgcr8* were conditionally ablated in the embryonic mouse neocortex, reported phenotypes which were often dominated by apoptosis, and massive tissue disorganization already at early stages of development (see for review Yang and Lai, 2011; Barca-Mayo and De Pietri Tonelli, 2014; Petri et al., 2014). This left unclear which of the phenotypes observed were due to loss of gene function, or secondary effects due to massive derangement of VZ/SVZ structure and NPC polarity (Arai and Taverna, 2017).

Here, we aimed to understand whether the increased generation of TBR1+ neurons and premature consumption of NPC pools resulting after conditional ablation of *Dgcr8* in the embryonic mouse neocortex was a secondary or reflected a direct consequence of DGCR8 loss of function (Marinaro et al., 2017). For this purpose, we overexpressed DGCR8 in the embryonic mouse neocortex. This resulted in a mosaic model, in which we found largely complementary phenotypes compared to our previous study (Marinaro et al., 2017), in absence of apoptosis (Figure 2 and Figure S2). Specifically, overexpression of DGCR8 in embryonic mouse neocortex reduces the generation of TBR1+ neurons and expands NPC pools (present study), while conditional knockout of *Dgcr8* increased generation of TBR1+ neurons and induced premature consumption of NPCs (Marinaro et al., 2017). Taken together, this evidence indicates that DGCR8 promotes cortical NPC self-renewal and represses their differentiation *in vivo*, possibly by a cell-autonomous function. Our results are consistent with previous observations in mouse ESCs (Wang et al., 2007; Cirera-Salinas et al., 2017a,b) and NPCs *in vitro* (Liu et al., 2017). Of note, overexpression of DGCR8 at later developmental stages (i.e., when upper cortical layer neurons are generated) selectively promotes expansion of BPs (Figure 5), opening intriguing perspectives for a DGCR8-dependent control in the radial neocortex enlargement in evolution, which reflects a striking increase in BP population and upper cortical layers size (Fietz and Huttner, 2011; Reillo et al., 2011; Shitamukai et al., 2011; Wang et al., 2011; Borrell and Reillo, 2012; Hevner and Haydar, 2012; Kelava et al., 2012; Betizeau et al., 2013; LaMonica et al., 2013).

On the other hand, the effects of DGCR8 overexpression on NPC proliferation (Figure 5) show distinct phenotypes compared to our previous study (Marinaro et al., 2017), and thus are less straight forward to interpret. For example, conditional deletion of *Dgcr8* led to decreased BrdU incorporation in BPs (Marinaro et al., 2017), while the proportion of BrdU+ BPs remained unaltered upon DGCR8 overexpression (present study). These differences could be due to the method used to label cell proliferation (BrdU incorporation), for instance BPs which undergo just one additional proliferative division might not be detected (Figure 5), or different developmental time dependent functions of DGCR8 (compare effects of NPC pools Figures 4, 6). Another possibility is that DGCR8 might simply repress neurogenesis in BPs, so that more electroporated cells remain “progenitors,” without changing proliferation index. Thus, despite our results support a model in which DGCR8 cell-autonomously promotes NPC expansion and represses neurogenesis, they did not provide conclusive evidence on the effect of DGCR8 on NPC proliferation.

Beside the well-known mechanism of DROSHA/DGCR8 Microprocessor complex in miRNA biogenesis *in vitro* (Ha and Kim, 2014) and *in vivo* (Yang and Lai, 2011; Barca-Mayo and De Pietri Tonelli, 2014; Petri et al., 2014), accumulating evidence indicates that these proteins also have alternative miRNA-independent functions (Burger and Gullerova, 2015). Indeed, DROSHA targets evolutionary conserved hairpin structures in mRNAs including *Dgcr8* itself, *Ngn2, Nf1a*, thereby regulating post-transcriptionally their expression independent of miRNAs (Han et al., 2009; Kadener et al., 2009; Shenoy and Blelloch, 2009; Karginov et al., 2010; Knuckles et al., 2012; Rolando et al., 2016; Kim et al., 2017; Marinaro et al., 2017). Similarly, we recently found that DGCR8 targets hairpins in *Tbr1* mRNAs. Thereby, DGCR8 represses *Tbr1* expression both at RNA and protein level (Marinaro et al., 2017). Other studies indicated that DGCR8 has also important functions in the regulation of splicing (Cirera-Salinas et al., 2017a,b). Thus DROSHA/DGCR8 alternative functions allow a fast regulation of the transcriptome and proteome, which might be crucially involved in the control of NPC maintenance and differentiation. However, the mechanisms and targets of DGCR8-dependent regulation in corticogenesis are still largely unknown. Here, we found that 3- to 5-fold increase of the DGCR8 level in electroporated cortices (Figures 1, 7), does neither change total levels of DROSHA, TDP-43, FOXG1, and DDX5 (Figure 7), nor the composition of the Microprocessor complex, with regard to the proteins that co-immunoprecipitated with DROSHA (Figure 7), nor the “miRNA-independent Microprocessor” functions, as revealed by similar levels of NGN2 protein expression (Figure 7). These results therefore suggest that DGCR8 might not necessarily engage in the DROSHA-Microprocessor complex to exert its functions in cortical NPCs. This hypothesis is consistent with *in vitro* data showing that human DGCR8 controls the stability of small nucleolar RNA (snoRNA) and other transcripts independently of DROSHA (Macias et al., 2015)

In conclusion, our results demonstrate that DGCR8 is essential for proper cortical development, and indicate that DGCR8 functions control NPC pool maintenance and neurogenesis, independently of DROSHA-Microprocessor complex. This is also in agreement with a recent study showing that DGCR8 mediates repair of UV-induced DNA damage independently of RNA processing (Calses et al., 2017). Intriguingly, DNA repair has been previously proposed to be involved in the maintenance of NPC pools (Arai et al., 2011). Future studies will be needed to demonstrate whether DGCR8-DNA repair pathway is causally involved in the maintenance of the NPC pools in corticogenesis.

## Materials and methods

### Mouse lines

Mice were housed under standard conditions at the animal facility of Istituto Italiano di Tecnologia (IIT), Genoa, Italy. All experiments and procedures were approved by the Italian Ministry of Health (Permits No. 057/2013; and 214/2015-PR –ref. IIT N° 071) and IIT Animal Use Committee, in accordance with the Guide for the Care and Use of Laboratory Animals of the European Community Council Directives. For *Dgcr8* cKO experiments *Emx1*-Cre^+/−^ (Iwasato et al., 2000) and *Dgcr8*^flox/flox^ (Yi et al., 2009) mice were crossed, genotyped and Cre-dependent *Dgcr8* deletion were ascertained as previously published (Marinaro et al., 2017). CD1 WT females and C57Bl6/J WT males were crossed, and embryos used for *in utero* electroporation experiments at the indicated days post coitum (dpc). For all time-mated animals vaginal plug day was defined as E 0.5.

### Plasmid cloning and *in utero* electroporation

Full length *Dgcr8* ORF (mmu-*Dgcr8* coding region NCBI Gene ID: 94223) was PCR amplified and cloned into pCAGGS vector (Niwa et al., 1991, modified in Clovis et al., 2012) with NheI and EcoRI. Primers used for *Dgcr8* amplification: forward: GGTCGGTGAGGGTCGACCGG and reverse: TTTATGTGTTCAGACCATCA.

*In utero* electroporation was performed as previously described (De Pietri Tonelli et al., 2006) with pCAGGS-mCherry/pCAGGS-mmu-*Dgcr8* (1:1 ratio, at 1 mg/ml, total concentration) or control pCAGGS-mCherry plasmids (at 1 mg/ml concentration). Cloning details for pCAGGS-mmu-DGCR8 plasmid are available upon request. Embryos were either immediately used (protein extraction) or fixed in 4% paraformaldehyde in Phosphate-buffered saline (PBS) at 4°C overnight (for immunofluorescence).

### BrdU labeling, immunofluorescence and imaging

BrdU labeling was carried out by 3 intraperitoneal injections, performed at 2-h intervals, of pregnant females at the indicated dpc (average mouse weight, 22–24 g), using 1 mg of BrdU (Sigma-Aldrich B5002-5G) in PBS, per injection. Mice were sacrificed 10 h after first BrdU injection [as previously performed (Marinaro et al., 2017)]. Coronal cryosections (20 μm) through brains (post-fixed in 4% PFA (paraformaldehyde; Sigma-Aldrich) and de-hydrated in 30% Sucrose) were prepared at the indicated ages, and processed for immunofluorescence. Immunofluorescence was performed as in Marinaro et al. (2017). Briefly, re-hydrated cryosections (subjected to antigen retrieval with 10 mM citric acid at pH 6.0 for 10 min at 95°C or 30 min at 80°C, if stained for BrdU), were permeabilized with progressive steps in 0.3 and 0.1% Triton X-100 in 1x PBS (PBST). For BrdU labeling 30 min incubation at 32°C in HCl 2N was performed prior to permeabilization, followed by blocking in 0.1% PBST + 5% normal goat serum for 1 h. Sections were afterwards incubated with primary antibodies: rabbit monoclonal anti-DGCR8 (Abcam, ab191875, 1:100), rabbit polyclonal anti-TBR1 (Abcam, ab31940, 1:200), rabbit polyclonal anti-TBR2 (Abcam, ab23345, 1:400), rabbit polyclonal anti-PAX6 (Covance, PRB2789, 1:500), mouse monoclonal anti-NGN2 (R&D, MAB3314, 1:500), rat monoclonal anti-BrdU (Abcam, ab6326, 1:200), rabbit polyclonal anti-KI67 (Abcam, ab15580, 1:250), rabbit monoclonal anti-CASPASE-3 (Cell Signaling, #9664, 1:400), rabbit polyclonal anti-SOX5 (Abcam, ab94396, 1:500), rabbit polyclonal anti-CUX1 (Santa Cruz, SC13024, 1:100) diluted in blocking solution overnight at 4°C in the darkness. Afterwards extensively washed in 0.1% PBST and incubated with secondary antibodies (Thermofisher: goat polyclonal anti-rabbit Alexa Fluor®488 (A-11034, 1:1000), goat polyclonal anti-rabbit Alexa Fluor®647 (A32733, 1:1000), goat polyclonal anti-mouse Alexa Fluor®488 (A32723, 1:1000) and goat polyclonal anti-rat Alexa Fluor®647 (A-21247, 1:1000), goat polyclonal anti-rabbit) diluted in blocking solution for 2 h at RT. Progressive washing steps in 0.1% PBST and then 1x PBS were performed, and sections were incubated with Hoechst (1:300 in 1x PBS from a stock solution of 1 mg/ml in dimethyl sulfoxide, DMSO, Sigma) for 30 min in the darkness, extensively washed in 1x PBS, mounted with ProLong Gold Antifade (Invitrogen), air-dried overnight in the darkness, and sealed with nail polish (Electron Microscopy Sciences). Fluorescent images were acquired with Nikon A1 using a 20x or 60x objective and analyzed with Nikon software version 4.11.0 (NIS Elements Viewer) and ImageJ version 1.48v (Wayne Rasband, National Institutes of Health, USA).

### Analysis of embryonic dorsal telencephalon immunofluorescence images

Immuno-positive cells for the indicated markers were counted through the depth of the telencephalic wall in the electroporated area and their numbers expressed as a proportion of total number of electroporated cells as indicated in figures and legends. For all the presented quantifications, all relevant sections containing electroporated cells from rostral to caudal were quantified upon DGCR8 overexpression and control conditions. Images represented in Figures 3, 4, 5B, 6C,E, 7 show the maximum projection of 10 μm z-Stack acquisitions. Images represented in Figures 1, 2, 5G, 6A,A' and Figures S1, S2 show single z-section acquisitions.

### Western blotting

For total protein extraction, electroporated areas of embryonic neocortices were homogenized in RIPA buffer (NaCl 3M, Triton X-100, Sodium Deoxycholate 0.5%, SDS 10%, TrisHCl 1M) supplemented with protease inhibitor (1 tablet protease inhibitor cocktail, 7x, after manufacture's instructions, Roche) and SOV (sodium orthovanadate, 1 mM, Sigma-Aldrich). Tissue was sonicated (10 short pulses, Branson Sonifier 150, Remote, Programm 1) and left on ice for 15 min. Lysate was clarified by centrifugation at 17949 × g for 30 min at 4°C. Protein concentration was determined by using the Bradford Assay kit (Bio-Rad) with a photospectrometer (Eppendorf; BioSpectrometer). For blot analysis, equal amounts of denatured protein (5 min at 100°C) were run on Mini-PROTEAN_TGXTM Precast Gels (Bio-Rad) and transferred on nitrocellulose membranes (GE Healthcare). Membranes were blocked in 5% milk powder in 0.2% PBS-Tween-20 for 1 h at RT, probed with rabbit polyclonal anti-DGCR8 (Proteintech, 10996-1-AP, 1:1000), rabbit polyclonal anti-SOX5 (Abcam, ab94396, 1:1000) and rabbit anti-ACTIN (Sigma, A2066; 1:5000) overnight at 4°C, followed by incubation with HRP-conjugated secondary antibody anti-rabbit (Invitrogen, A16104; 1:2000) for 2 h at RT. For all wash steps 0.2% PBS-Tween-20 was used. LAS 4000 Mini Imaging System (GE Healthcare, Little Chalfont, UK) was used for detection of chemiluminescence using SuperSignal® West Pico reagent (ThermoScientific). Band intensities were quantified using ImageJ.

### Co-immunoprecipitation

For total protein extraction, electroporated areas of embryonic neocortices were lysed in Co-IP buffer (100 mM NaCl, 20 mM Tris, 1 mM EDTA, 0,5% NP-40) supplemented with complete Protease Inhibitor Cocktail (Roche-Diagnostics) for 30 min on ice and triturated with a 1 ml pipette every 10 min 20 times. Lysate was clarified by centrifugation at 17949 × g for 10 min at 4°C and the supernatant was collected. Protein concentration was determined by using the Bradford Assay kit (Bio-Rad) with a spectrophotometer (BioPhotometer, Eppendorf). Equal amounts of protein was used for all MOCK and Co-IPs, 5% was used for the input. Protein G Dynabeads (10004D, ThermoScientific) were coupled with rabbit polyclonal anti-DROSHA (Abcam, ab12286, 1:100) or rabbit-IgG (rabbit IgG kch-504-250, Diagenode, Seraing, Belgium) in Co-IP buffer under rotation for 1.5 h at RT and 1 h at 4°C. Tissue lysates were precleared with Protein G Dynabeads in Co-IP buffer under rotation for 1 h at 4°C. Subsequently tissue lysates were transferred to antibody-coupled beads and incubated while rotating at 4°C overnight. Beads were washed 3 times with 1 ml Co-IP buffer before they were re-suspended in 30 μl 1x Laemmli buffer (4% SDS, 20% glycerol, 10% 2-mercaptoethanol, 0.004% bromphenol blue and 0.125 M Tris HCl, pH approximately 6.8). For immunoblotting equal amounts of denatured proteins (5% input and the complete Co-IP samples, 5 min at 95°C) were used. Protein and Co-IP samples were loaded on 10% SDS-polyacrylamide gels and run for 1.5 h at 120 V. Proteins were transferred to PVDF membranes (Trans-blot Turbo Transfer Pack) using the Trans-blot Turbo System (Bio-Rad) following the manufacturer's instructions. Membranes were blocked with 5% BSA or 5% milk powder in 0.1% TBS-Tween-20, probed with rabbit polyclonal anti-DDX5 (Abcam, ab126730, 1:2000), rabbit polyclonal anti-FOXG1 (Abcam, ab18259, 1:1000), rabbit monoclonal anti-DGCR8 (Abcam, ab191875, 1:1000), rabbit polyclonal anti-TDP-43 (Proteintech, 10782-2-AP, 1:5000) and mouse monoclonal anti-GAPDH (Abcam, ab8245, 1:3000). Followed by incubation with HRP-conjugated secondary antibody anti-rabbit (1:10000, donkey-anti-rabbit, 111-035-003, Dianova) or anti-mouse (1:10000, donkey-anti-mouse, 115-035-003, Dianova) for 1 h at RT. For all wash steps 0.1% TBS-Tween-20 was used. LAS 4000 Mini Imaging System (GE Healthcare, Little Chalfont, UK) was used for detection of chemiluminescence using Femto substrates (Thermo Scientific). Band intensities were quantified using ImageJ.

### Statistical analysis

Experimental numbers (n) in Figure 1, and 3–7 (immunostaining) are cortices from independent mouse embryos from at least 2 independent litters; while each “n” in Figure 7 (Co-IP experiments) is a pool of 5–6 extracts from electroporated cortical areas. Data are expressed as standard error mean (s.e.m.) for all quantifications and assays. Differences between groups were tested for statistical significance, where appropriate using unpaired Student's *t*-test or two-way ANOVA followed by Tukey's *post hoc* testing. Significance was expressed as follows in all figures: ^*^p-value < 0.05; ^**^p-value < 0.01; ^***^p-value < 0.001; n.s.: not significant.

## Author contributions

DD conceived, supervised and coordinated the project. FM co-supervised NH during initial experiments. Investigation: NH performed all the experiments and analyzed data. SW carried out the experiment in Figures 7A–C under TV supervision. Visualization: NH prepared all the figures. DD and NH co-wrote the manuscript. All authors approved the final version of the manuscript.

### Conflict of interest statement

The authors declare that the research was conducted in the absence of any commercial or financial relationships that could be construed as a potential conflict of interest.
