# Supplementary figures and images for: DGCR8 Promotes Neural Progenitor Expansion and Represses Neurogenesis in the Mouse Embryonic Neocortex
Source: Front Neurosci. 2018 Apr 30;12:281. doi: 10.3389/fnins.2018.00281 (PMC5936999; doi:10.3389/fnins.2018.00281)

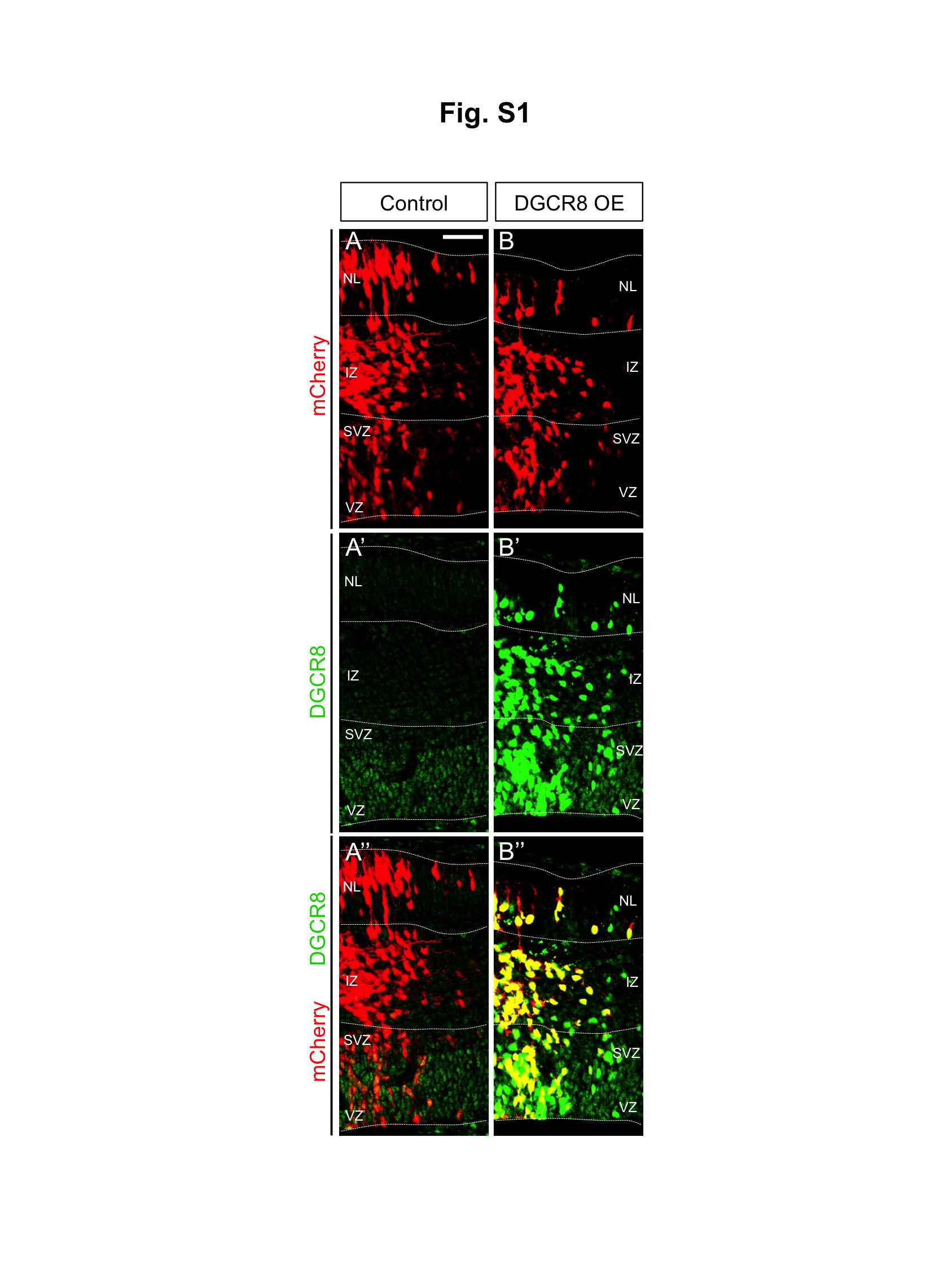

Supplement: Supplementary file 1 [file Image_1.TIFF]

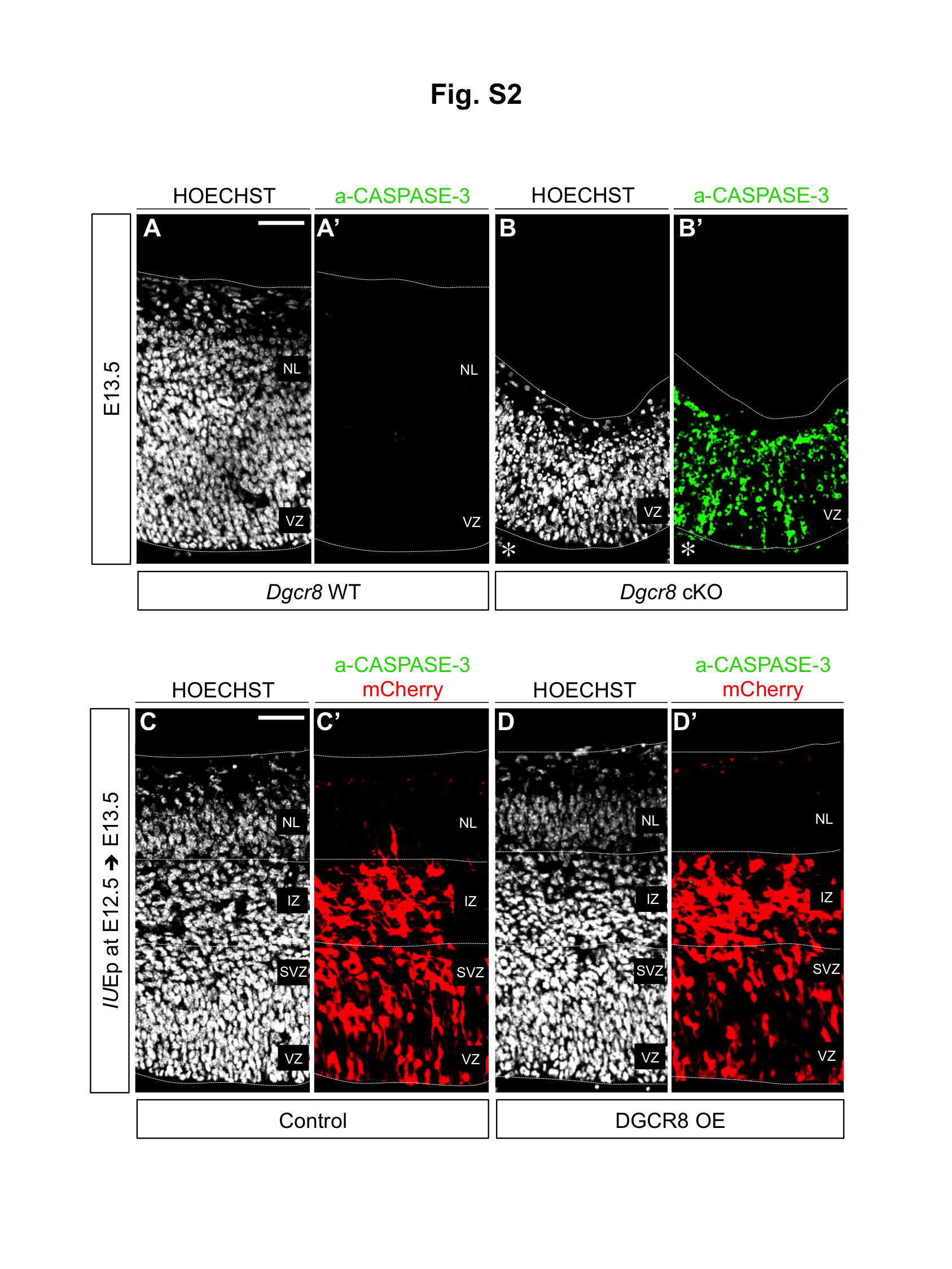

Supplement: Supplementary file 2 [file Image_2.TIFF]

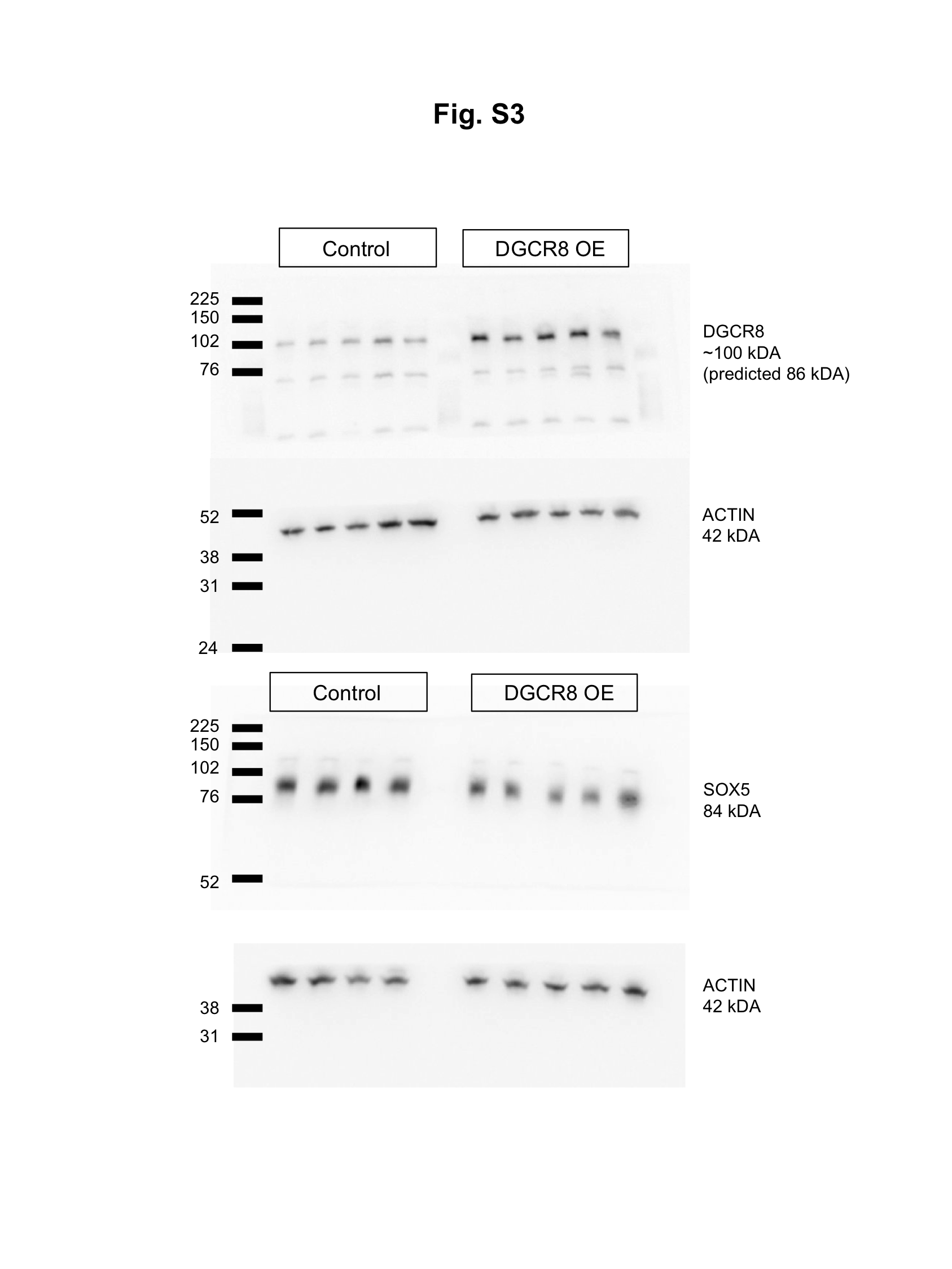

Supplement: Supplementary file 3 [file Image_3.TIFF]

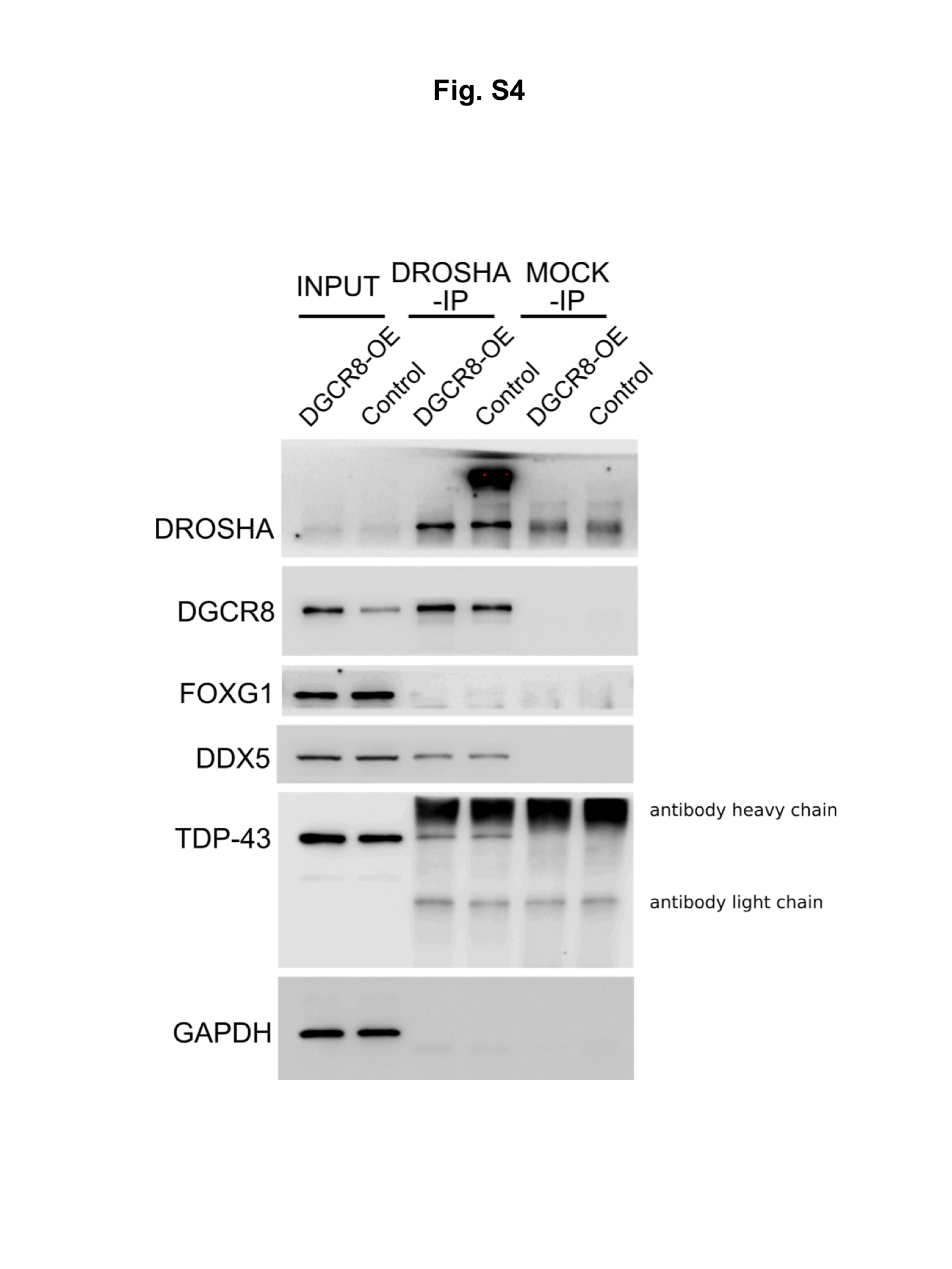

Supplement: Supplementary file 4 [file Image_4.TIFF]
